# The effect of national protest in Ecuador on PM pollution

**DOI:** 10.1038/s41598-021-96868-6

**Published:** 2021-09-02

**Authors:** Rasa Zalakeviciute, Katiuska Alexandrino, Danilo Mejia, Marco G. Bastidas, Nora H. Oleas, Diana Gabela, Phuong Ngoc Chau, Santiago Bonilla-Bedoya, Valeria Diaz, Yves Rybarczyk

**Affiliations:** 1 Grupo de Biodiversidad Medio Ambiente y Salud (BIOMAS), Universidad de Las Américas, calle José Queri y Av. de los Granados / Bloque 7, 170125 Quito, EC Ecuador; 2 Facultad de Ciencias Químicas de La Universidad de Cuenca, Cuenca, Ecuador; 3 Centro de Estudios Ambientales (CEA) de la Universidad de Cuenca, Cuenca, Ecuador; 4 Centro de Investigación de la Biodiversidad y Cambio Climático (BioCamb) e Ingeniería en Biodiversidad y Recursos Genéticos, Facultad de Ciencias de Medio Ambiente, Universidad Tecnológica Indoamérica, Machala y Sabanilla, 170301 Quito, EC Ecuador; 5 Faculty of Data and Information Sciences, Dalarna University, 791 88 Falun, Sweden; 6 Research Center for the Territory and Sustainable Habitat, Universidad Tecnológica Indoamérica, Machala y Sabanilla, 170301 Quito, Ecuador; 7 Secretariat of the Environment, Quito, Ecuador

**Keywords:** Environmental sciences, Engineering

## Abstract

Particulate matter (PM) accounts for millions of premature deaths in the human population every year. Due to social and economic inequality, growing human dissatisfaction manifests in waves of strikes and protests all over the world, causing paralysis of institutions, services and circulation of transport. In this study, we aim to investigate air quality in Ecuador during the national protest of 2019, by studying the evolution of PM_2.5_ (PM ≤ 2.5 µm) concentrations in Ecuador and its capital city Quito using ground based and satellite data. Apart from analyzing the PM_2.5_ evolution over time to trace the pollution changes, we employ machine learning techniques to estimate these changes relative to the business-as-usual pollution scenario. In addition, we present a chemical analysis of plant samples from an urban park housing the strike. Positive impact on regional air quality was detected for Ecuador, and an overall − 10.75 ± 17.74% reduction of particulate pollution in the capital during the protest. However, barricade burning PM peaks may contribute to a release of harmful heavy metals (tire manufacture components such as Co, Cr, Zn, Al, Fe, Pb, Mg, Ba and Cu), which might be of short- and long-term health concerns.

## Introduction

Particulate matter (PM) has become one of the leading causes of human health problems around the world, often due to the toxicity of its chemical composition. Consequently, these micro-sized air pollutants are responsible for millions of premature deaths each year, attributable to the damage imposed on the respiratory and cardiovascular systems^1–3^. PM can originate from various sources, such as traffic, thermoelectric plants, oil refineries, fires, among others^4^. Thus, the concentration and composition of PM at a specific location depend on an array of factors, such as the characteristics of local sources, regional context, weather conditions and changes in human activities.

Human dissatisfaction, due to social unrest, inequality or implementation of certain regulations, laws or policies can trigger protests and even strikes. As an example, over the course of 2019, a huge number of protests developed all over the world including Asia, Europe and the Americas^5–8^. At the end of the year, Latin America was the main stage of demonstrations against governments in Mexico^9^, Chile^10^, Bolivia^11,12^, Colombia^13^ and Ecuador^14^. Massive demonstrations and severe riots flared up in the capital cities and in some cases spread all over the country, lasting from a few weeks (Ecuador, Bolivia) to months (Chile, Mexico, Nicaragua, Colombia) and even spilling into the next year (Argentina, Peru, Colombia, Nicaragua and Mexico)^15,16^.

Strikes can often be characterized by reduced function of institutions or services, and a substantial reduction of vehicle circulation, causing a decrease in emissions^17,18^. However, to paralyze the area, the demonstrations could even involve blocking roads by barricading or burning different materials, such as wood, tires, etc. Unfortunately, smoke from the combustion of these materials can contain hundreds of different chemical elements and compounds, many of which are harmful to health and potentially carcinogenic and mutagenic^19–22^. For example, pollutants in wood smoke, which is often used to alleviate the eye-burning effect of tear gas^23^, includes nitrogen oxides, sulfur oxides, carbon monoxide, volatile organic compounds and fine particles saturated with toxic metals^24–26^. On the other hand, vehicle tire smoke—a common protest tool in Latin America due to its black smoke—can also contain a large amount of highly toxic metals used in its manufacture^27,28^. These metals, such as copper, iron, magnesium, barium and zinc, are released into the atmosphere as particles. They are not chemically and biologically degraded, making them stable and persistent in the environment. This raises a concern for a rapid deterioration in air quality during those events.

While there are a number of social studies about the reasons and impacts of strikes^29,30^, there is a very limited amount of research on air quality impact. A study in Hong Kong reported a reduction in pollutant emissions from traffic (substantially reduced NO_2_ and just slightly reduced PM in the area of the protests) during large-scale street protests^7^. In another study focusing on truck drivers’ strike impact on air pollution in São Paulo, it was observed that while the concentrations of CO and NO decreased by 50%, NO_2_ and PM showed almost no changes, suggesting the relevance of secondary reactions and other sources, such as fires^31^. Studies carried out in Italy and Spain during public transport strikes indicated that the concentration of pollutants, such as CO, increased due to a greater use of private vehicles^32,33^. These contradictions in the existing research detain us from understanding the general impact of protests on regional and even global air quality. The research carried out worldwide on the impacts of the strikes on air pollution has investigated the concentration of gases and particulate matter. However, to the best of our knowledge, there is no information on the concentration of chemical elements in the particulate matter or other material, which might pose a serious threat to human health.

In light of the rise of global social dissatisfaction, it is important to understand the broader environmental impact of strike events. Therefore, this investigation aimed to assess air quality in Quito, Ecuador, during the national strike of October 2–13, 2019. During the strike, there was an absence of urban buses and a great reduction in the use of private vehicles because the schools, universities and other services stopped their activities completely. This shaped a natural experiment allowing us to assess the impact of transportation over PM emissions in the city. Thus, the strike impact on regional and local air quality was evaluated using satellite and ground-based monitoring data. Then, the evolution of PM_2.5_ (an aerodynamic diameter ≤ 2.5 μm) concentrations during this event was evaluated all over the city using machine learning modeled business-as-usual conditions set against the real data. Finally, since emissions of metals into the urban airshed can occur during the burning of protest objects such as tires, causing negative implications to human health, a chemical analysis of plant samples from the urban park, where the protests were centered, was carried out to determine the presence of toxic metals. Plants, as indicators of air pollution, have been widely used around the world, because pollutants can be retained in their organs or tissues (e.g., leaves)^34–36^. This allowed detecting the presence of toxic metals at the point of interest^37^. This study, therefore, can help us understand the impact of the strike on air quality at different urban scales.

## Results and discussion

### Satellite PM_2.5_ concentration data

**Figure 1:**
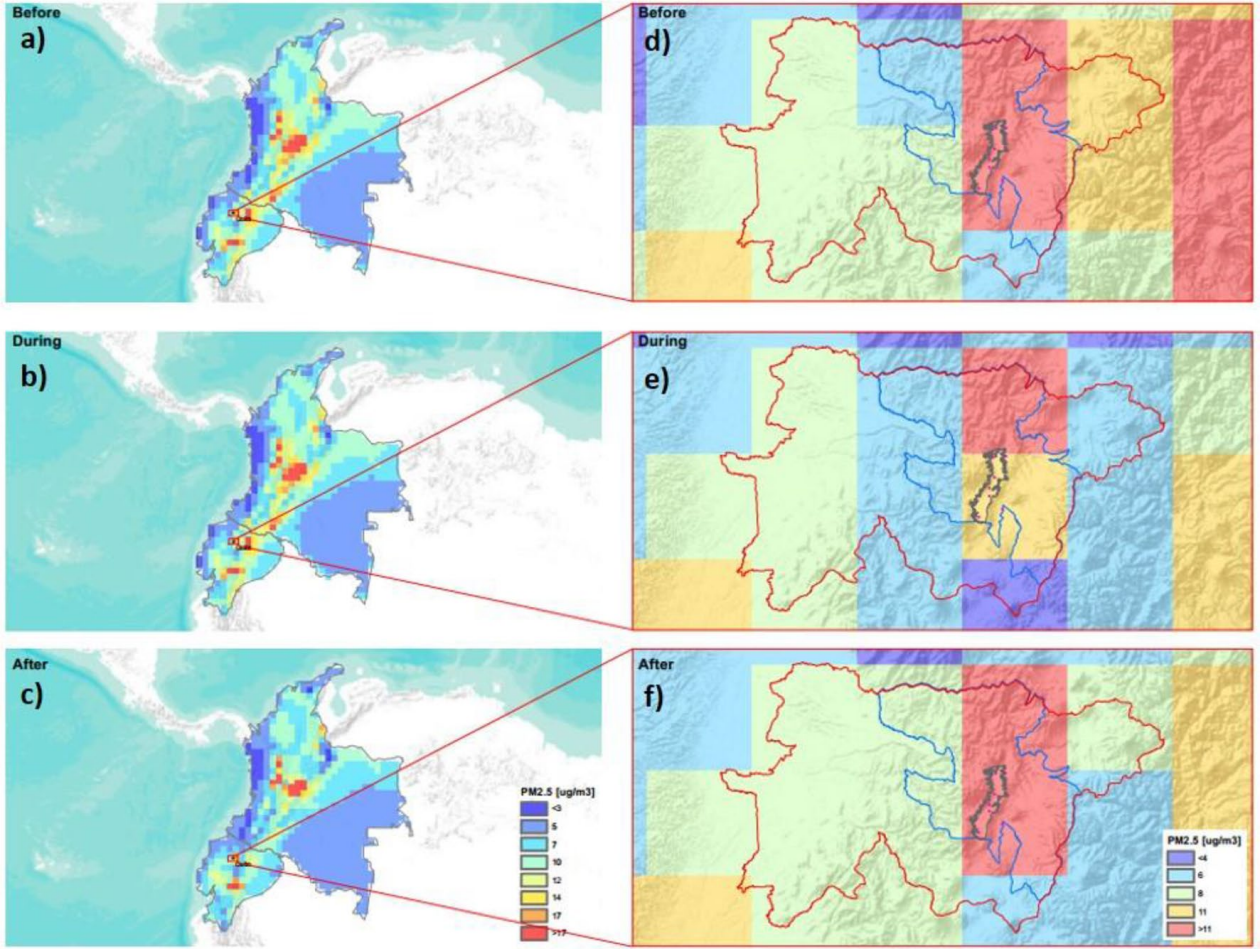
Satellite images of PM_2.5_ concentrations for Ecuador and Colombia (panels **a**–**c**) and the Pichincha region (red line) of Ecuador, containing Metropolitan District of Quito (blue line), and Quito city (grey line) (panels **d**–**f**) averaged over 12 days of three periods: September 25–October 1 (before the strike, panels **a** and **d**), October 2–13 (during the strike, panels **b** and **e**) and October 14–25 (after the strike, panels **c** and **f**). For the visualization of the ECMWF raster of PM_2.5_ the ArcGis PRO software was used, with a resolution of 44 km /pixel, with a WGS84 projection system with ESPG 4326 coordinate system , these data are available at https://developers.google.com/earth-engine/datasets/catalog/ECMWF_CAMS_NRT?hl=en.

The national Ecuadorian strike of 2019 lasted 12 days during October 2–13, drawing people from all over the country to the capital city Quito. First, to study the impact of the strike on PM_2.5_ levels in Ecuador, satellite images were analyzed for the whole country and the region (Fig. 1a–c). Comparing among the different 12-day periods (before (9/20/2019–10/1/2019, Fig. 1a), during (10/2/2019–10/13/2019, Fig. 1b) and after (10/14/2019–10/25/2019, Fig. 1c) the strike), it was observed that most of the polluting protest activities were present in the capital city Quito. Therefore, the focus of this study was narrowed down to Quito, the central place for the national strike. It was observed that Ecuadorian regional air quality during those dates was highly affected by the volcanic activity (active Sangay volcano ash plumes were detected in the Central part of the country, reaching over 70 µg m^−3^) (Fig. 1a–c). There were a few active-erupting volcanoes in the Andes cordillera at the time of the strike^38^. Also, a background PM pollution was spotted creeping in from the north (Colombia) along the eastern Andes cordillera (Fig. 1a–c). The mountains function as a barrier to push the pollution south along the mountain ridge, with the prevailing northeasterly winds.

Figure 1d–f show satellite images of PM_2.5_ concentrations for the Pichincha region (red line) of Ecuador, containing the city of Quito (gray line), averaged over the three studied periods. The average PM_2.5_ levels in the whole district of Pichincha during the strike were lower (6.99 µg m^−3^) than before (8.04 µg m^−3^) and after (7.8 µg m^−3^) the strike. The PM_2.5_ levels in two 45 km × 45 km cells of Quito clearly reduce during the strike (11.13 µg m^−3^) if compared to before (12.92 µg m^−3^) and after (14.37 µg m^−3^) the strike as well. This shows a 13.85% reduction during the protests, if compared to the levels before the strike. Overall, regional changes from the reduced urban activities are clearly visible near Quito (Fig. 1). This is expected, as one of the biggest polluting sources in Quito (and Ecuador) is a poor-quality fuel used by older technology motorized fleet^39^. Therefore, our findings point to a positive regional impact of protests with paralysis of motor vehicular circulation on the environmental air quality in Ecuador, and most likely in other South American countries.

### PM_2.5_ concentration change

**Figure 2:**
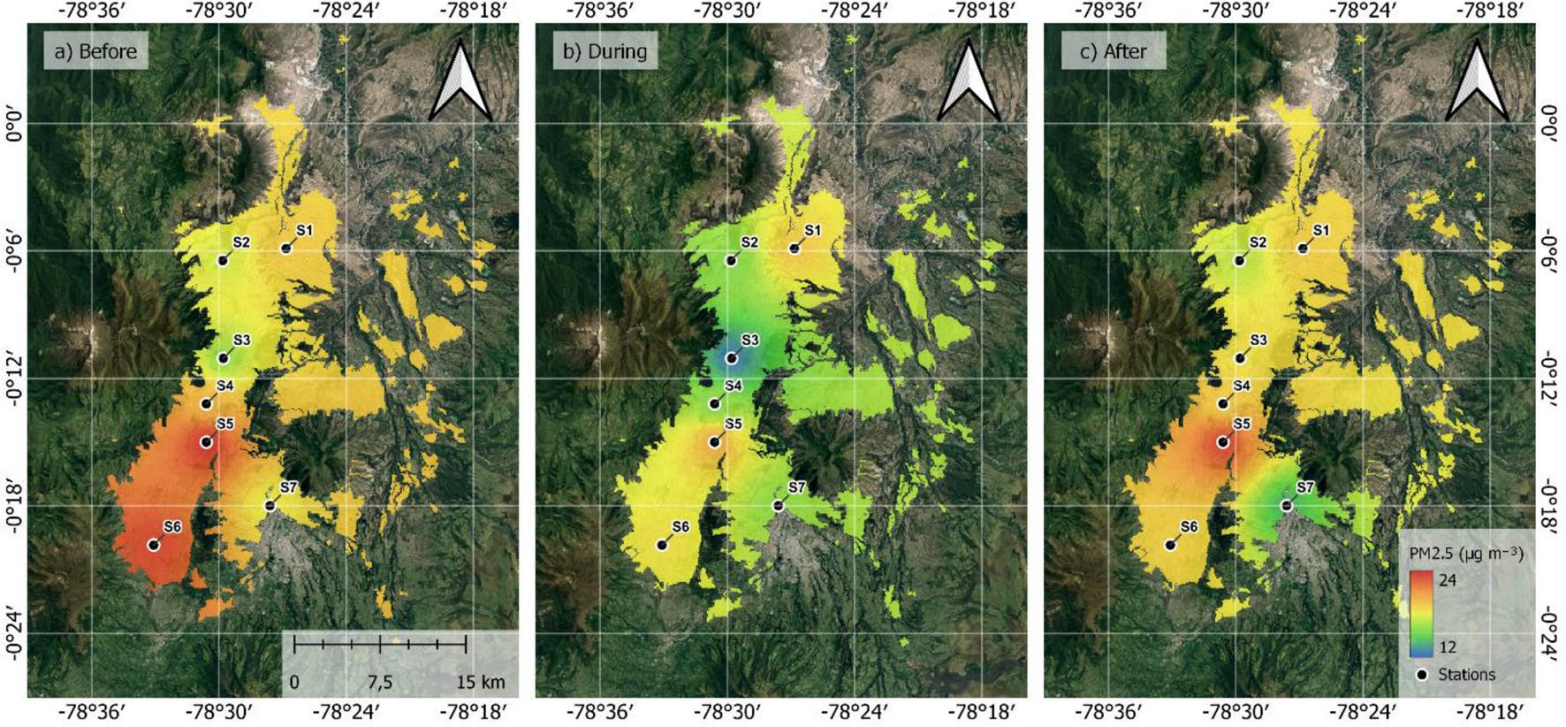
Inverse distance weighting interpolation of the average PM_2.5_ levels at the ground-based monitoring sites in the Ecuadorian capital Quito: (**a**) before (September 25–October 1), (**b**) during (October 2–13) and (**c**) after (October 14–25) the 2019 national strike. Geographical Information Software QGIS 3.18.2 was used ( available at https://qgis.org/downloads/), employing ESPG 4326 (WGS 84) (panels **a**–**c**) and Urban Growth of Quito was used for limits (retrieved from http://gobiernoabierto.quito.gob.ec/?page_id=1122).

While satellite data can provide an invaluable picture of the regional effects of strikes, they cannot possibly display the urban pollution in different parts of the city in greater detail. Hence, Table 1 shows statistics of surface level PM_2.5_ data for seven monitoring sites of Quito for the three 12-day periods: before (9/20/2019–10/01/2019), during (10/02/2019–10/13/2019) and after (10/14/2019–10/25/2019) the strike. Most of the sites show the highest PM_2.5_ levels before the strike, with an overall reduction of 13.9% during the strike. These results compare very well with the satellite data (Fig. 1). The same data can be visualized, in spatial interpolation graphs for each period in Fig. 2. It can be confirmed that most of the sites show a significant reduction of the PM_2.5_ concentrations during the protests. There are two exceptions. S1-Carapungo, the site in the northern outskirts of the capital, indicates a small increase in PM_2.5_ concentrations during the 12 days of strike. This increase might be due to the barricades and burning events near the entrances to the city in the outskirts. Meanwhile the central S3-Belisario site shows an increase in PM_2.5_ concentrations after the strike, which agrees with satellite data post-strike. Post-strike weather became rainier and more humid (Figure A1, Appendix 1), which might influence an increase in vehicle use and worsened combustion and thus increased anthropogenic PM_2.5_ emissions in busy traffic areas^40^. While these data might be helpful to estimate urban population’s exposure to air pollution, it might be skewed by the variation in meteorological conditions.

**Table 1:** Average PM_2.5_ concentration (µg m^−3^) change before (September 25–October 1), during (October 2–13) and after (October 14–25) the national 2019 strike in seven monitoring sites in Quito, Ecuador.

Site ID	Site name	Before 09/20–10/01	During 10/2–10/13	After 10/14–10/25
S1	Carapungo	19.33 ± 12.99	**19.36** ± 19.23	19.23 ± 15.39
S2	Cotocollao	**17.48** ± 10.29	15.96 ± 7.79	16.99 ± 9.97
S3	Belisario	16.24 ± 9.26	12.69 ± 7.18	**18.43** ± 15.39
S4	Centro	**19.95** ± 9.44	15.93 ± 10.33	19.12 ± 10.39
S5	Camal	**23.78** ± 15.06	20.29 ± 29.11	23.29 ± 16.00
S6	Guamani	**23.12** ± 17.68	18.45 ± 13.41	19.04 ± 11.38
S7	Chillos	**18.06** ± 9.72	16.13 ± 8.44	14.25 ± 8.28
Average	Quito	**19.71**	16.97 (-13.9%)	18.62 (-5.5%)

The biggest values out of the three periods are marked in bold font.

### PM_2.5_ concentration change: machine learning modelling

**Figure 3:**
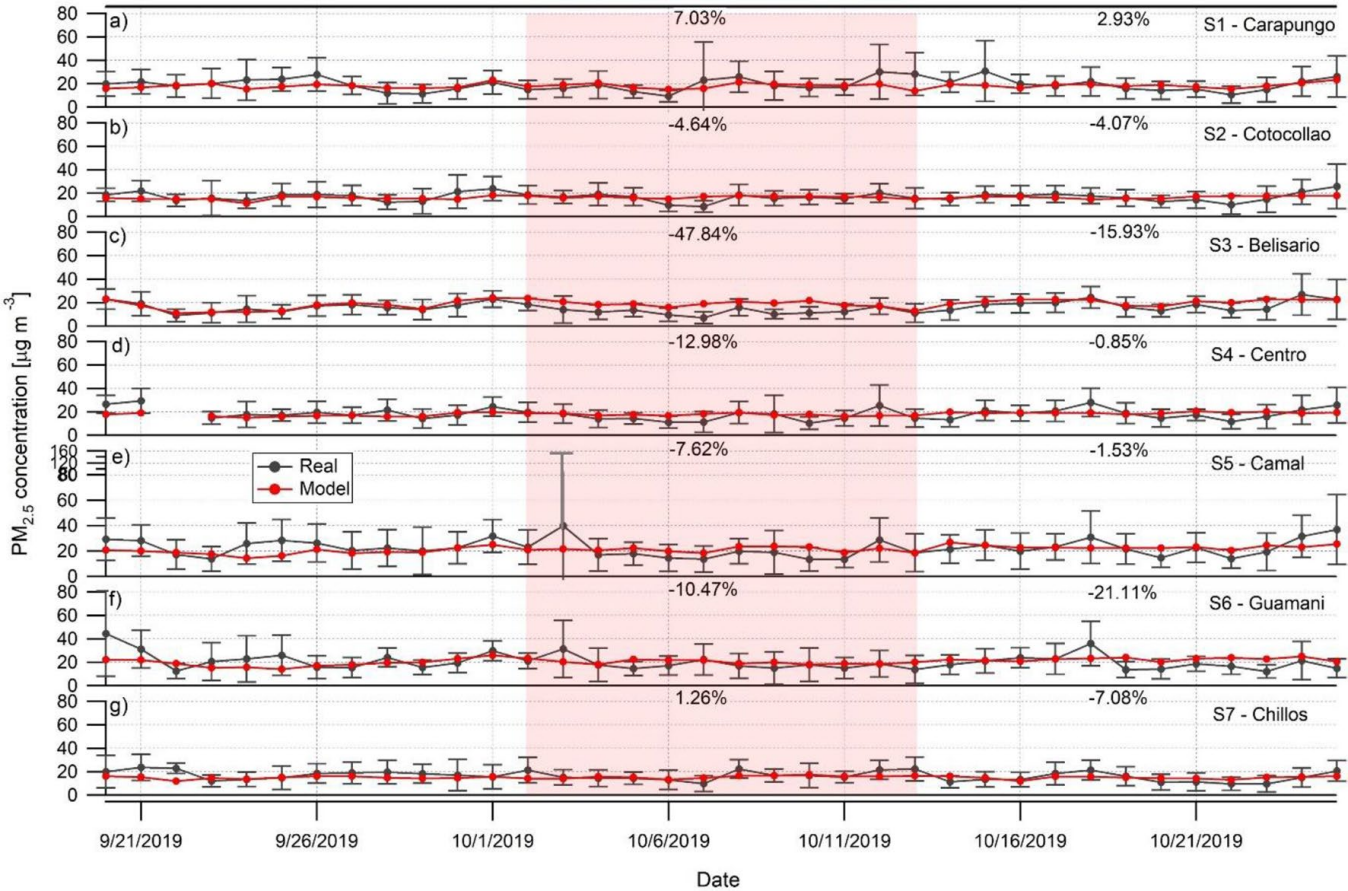
In black: daily averages and standard deviation (error bars) of PM_2.5_ concentrations; in red: modeled values for PM_2.5_ business-as-usual conditions September 20–October 25, 2019. The national strike took place on 2–13 of October 2019, identified by red shaded areas. Each panel represents different site across Quito from the most northern to the most southern district: (**a**) S1 – Carapungo; (**b**) S2 – Cotocollao; (**c**) S3 – Belisario; (**d**) S4 – Centro; (**e**) S5 – Camal; (**f**) S6 – Guamani; and (**g**) S7 – Chillos. The percentage values identify change (negative = reduction, positive = increase) in PM_2.5_ real concentrations from business-as-usual conditions.

To unbiasedly estimate the impact of the strike on PM_2.5_ levels, the real PM_2.5_ concentrations were compared to the business-as-usual PM_2.5_ concentrations predicted by ANN models (Fig. 3). This approach has been proven more accurate rather than comparing the pollution changes during the protest to the real data from another period^41^, since the estimated data are provided by meteorology-normalized models^42^. Before the strike, the validation of the model can be verified as the modeled data mostly overlap with the actual PM_2.5_ concentrations (the estimated values in Fig. 3 are within the standard deviation of the observed values). The predicted data (red line in Fig. 3) are obtained from the model getting the highest performance (see Table A1, Appendix 1, for the assessment of the modeling accuracy). Consequently, the business-as-usual air quality is determined by LSTM for S1-Carapungo, S3-Belisario, S5-Camal, S6-Guamani and S7- Chillos, and simple RNN for S2-Cotocollao and S4-Centro. The model accuracy is high for S2-Cotocollao, S3-Belisario, and S7-Chillos (RMSE < 10 µg m^−3^). The performance is good for S1-Carapungo, S4-Centro and S5-Camal (RMSE < 13 µg m^−3^), and only fair for S6-Guamani (RMSE = 15.6 µg m^−3^). Missing data in the training set can explain a lower accuracy for this latter model. Overall, the performance of the models is high enough to provide us with a reliable prediction of the business-as-usual air quality conditions.

It can be observed that daily PM_2.5_ concentrations decreased during the strike (− 10.75 ± 17.74%) over the whole Andean city if compared to business-as-usual (red shaded area in Fig. 3). This can be attributed to the limitations of traffic activities, due to the forced aggressive stagnation of any type of transportation. To confirm this, the sites that demonstrate the biggest reduction are S3-Belisario (− 47.84 ± 14.40%, Fig. 3c) and S4-Centro (− 12.98 ± 73.50%, Fig. 3d). These two sites are located in the central traffic-busy areas. However, S4-Centro is the site closest to the central protest activity at El Ejido park, thus showing a lower PM_2.5_ reduction than in S3-Belisario. The paralysis of the use of buses and private cars can justify the greatly reduced particulate pollution, previously also seen in another study in Quito related to the strict regulations to slow down COVID-19 infections^43^. However, in the areas around the main road blockages (Figure A2, Appendix 1), the overall elevated pollution levels and an increase in PM_2.5_ concentration variations can be perceived. The former can be identified in the northern and southern city outskirts near S1-Carapungo (7.03 ± 63.72%, Fig. 3a) and S7-Chillos (1.26 ± 69.74%, Fig. 3g). Meanwhile the increased pollution variation (standard deviation) can be observed in S1-Carapungo (7.03 ± 63.72%, Fig. 3a), S2-Cotocollao (− 4.64 ± 69.57%, Fig. 3b), S4-Centro (− 12.98 ± 73.50%, Fig. 3d), S5-Camal (− 7.62 ± 67.57%, Fig. 3e), S6-Guamani (− 10.47 ± 59.83%, Fig. 3f) and S7-Chillos (1.26 ± 69.74%, Fig. 3g)—the areas near the main roads housing the barricades (Figure A2, Appendix 1). Due to the complex geography of this Andean city, main roads often bottleneck at tunnels, bridges, serpentine-highways around numerous canyons and mountains. This last finding must be stressed, as most of the city had blocked private and highly polluting public transport circulation. Often, road blockages would have obstructions and active burning activity. As a result, during the strike, towers of smoke could be seen all over the city (Figure A3, Appendix 1). The smoke dispersion and fire activity might have been enhanced due to the fact that during the strike the accumulation of precipitation was very low (Figure A1, Appendix 1). This may help explain a high standard deviation of the PM concentrations in many sites of the city, as plumes of smoke would be transported to the monitoring sites. Our results indicate that even when most of the normal activities are seized in the urban area due to the protests, other types of unusual activities might keep worsening the air quality.

Furthermore, we investigated the particulate pollution evolution 2 weeks after the protests, in order to study the “return speed” of atmospheric pollution to the business-as-usual conditions. The overall change of pollution was − 6.8% averaged over 12 days after the protest. Interestingly, while some sites returned to “normal” levels (S1-Carapungo, S4-Centro and S5-Camal), the rest of the sites in this high elevation city show a continuous reduction in PM_2.5_ pollution levels even 12 days after the national strike. This could be due to the time lag to get back to normal activities, especially, transportation of products and merchandize. We want to point out the social behavior during these unusual events like stocking up on food and water. This might have influenced the reduction of certain anthropogenic activities (e.g. grocery shopping or local and international tourism, etc.) for some time after the strike. In addition, when the strike was announced, the population might have increased their shopping a few days before the protests start, which might be observed as a slight increase from the business-as-usual levels (Fig. 3b–f). A further study is required to investigate the time needed to go back to business-as usual pollution levels. However, an increase in PM_2.5_ concentrations can already be seen in most sites at the end of the study period (see Fig. 3b–g).

The meteorology normalized approach is essential to get a fine and accurate analysis of the long-range impact of the strike on air pollution. The naïve observation of the raw data shows a 11.9% increase of the PM_2.5_ concentration in Belisario after strike (Table 1). This value contradicts the 15.9% decrease estimated by the machine learning technique (Fig. 3). This difference can be explained by the fact that the meteorological conditions are taken into account in the ANN method but not in the observational study. Relative humidity (RH) and wind speed (WS) have a strong influence on the PM_2.5_ concentration in Quito^44^. The higher the RH, the higher is the concentration. On the contrary, WS tends to reduce the level of fine particulate matter in the atmosphere. In Belisario before strike, the WS was stronger (1.42 m/s), and the RH was lower (65.5%) than after strike (WS = 1.14 m/s; RH = 75.5%). It is consequently not possible to reveal the pollution reduction effect of the strike, because it is masked by the high value of meteorological parameters that negatively affect air quality (Figure A1, Appendix 1, for more details about the meteorological conditions). Since the machine learning modeling is meteorology normalized, the prediction of PM_2.5_ concentration for business-as-usual is not impacted by this artifactual underestimation.

### Plant sample chemical analysis

**Figure 4:**
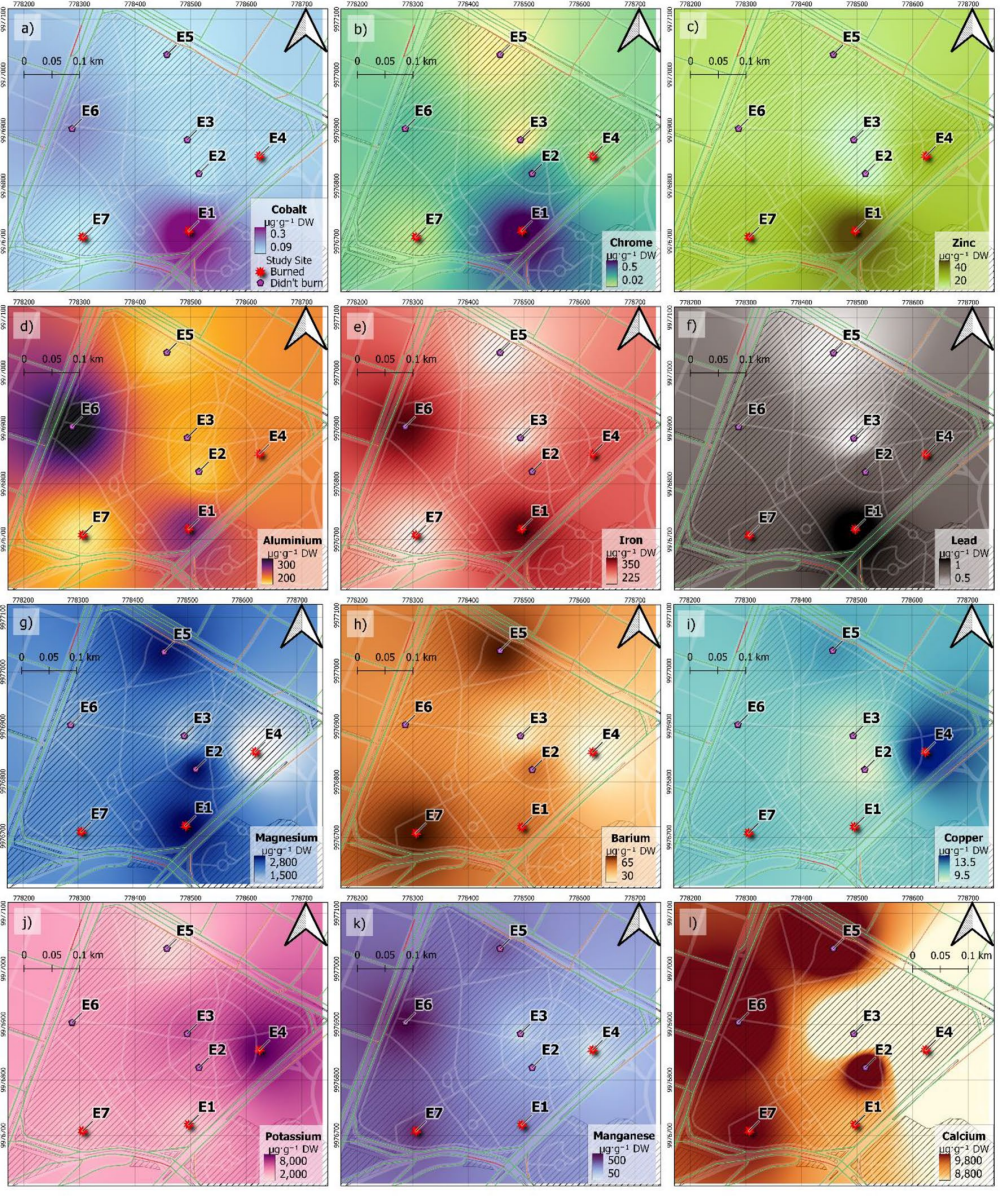
Distribution patterns of the concentration, μg g^−1^ DW, of metals found in the *Melaleuca armillaris* leaves collected at the urban El Ejido park two days after the national protests (October 15, 2019). Red markers: points with obvious burning signs at the moment of the sampling collection, E4 was marked with red marker as it is located just in front of a blockage of the road, where tires were burned. Red lines indicate common transects of traffic congestion (Google Maps Traffic). Geographical Information Software QGIS 3.18.2 was used ( available at https://qgis.org/downloads/), employing ESPG 4326 (WGS 84).

**Figure 5:**
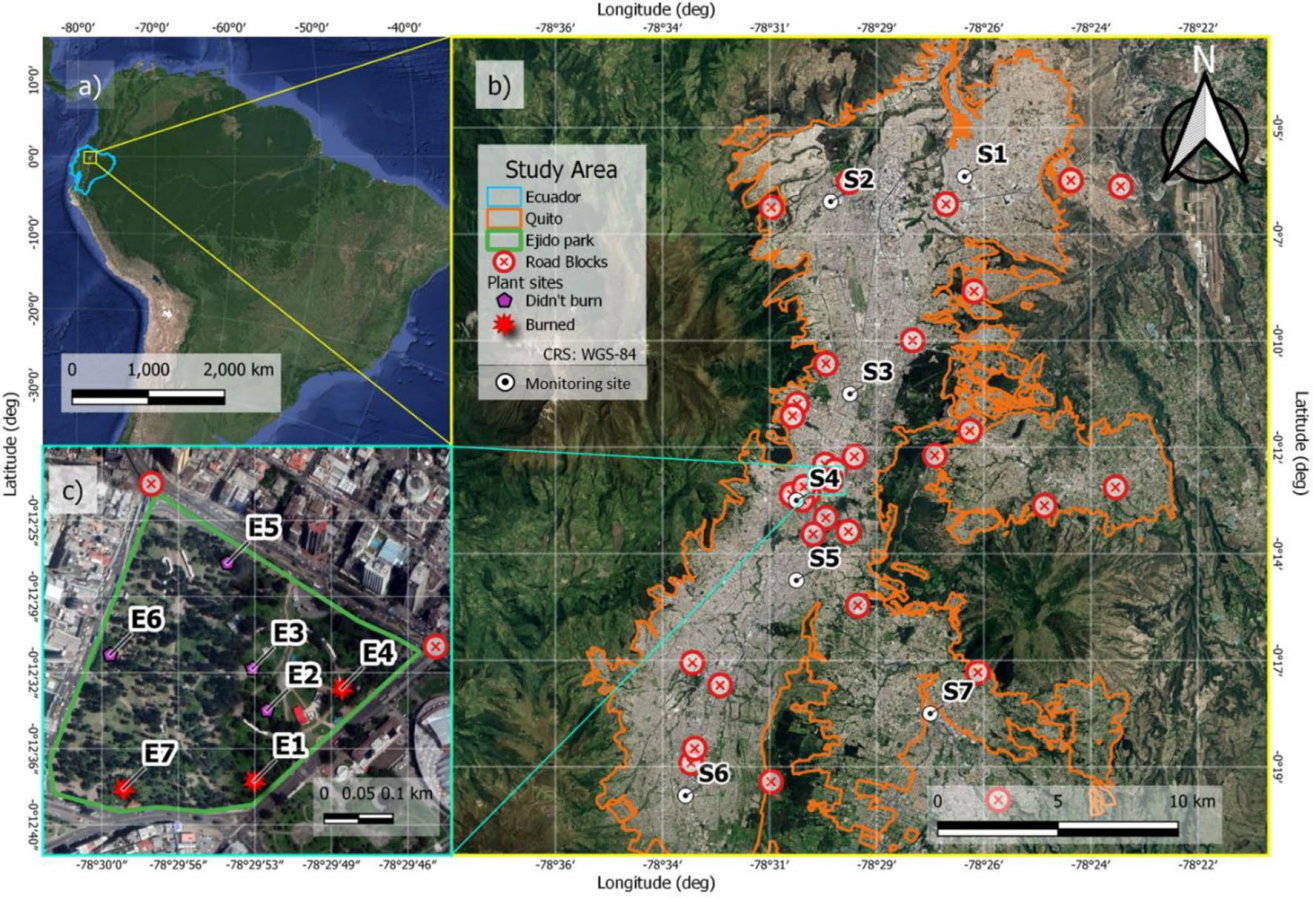
Study sites: (**a**) Map of the location of Ecuador in the context of South America; (**b**) Map of Quito and the selected PM_2.5_ measurement sites (S1–S7) belonging to the environmental monitoring network of the Secretariat of the Environment of DMQ; and c) *Melaleuca armillaris* leaves’ sampling sites (E1–E7) at El Ejido Park in the hot spot of the national protest. Spatial visualization software QGIS 3.18 was used, employing ESPG 4326 (WGS 84) (panel **a**) and EPSG.32717 (WGS84-UTM17 South) coordinate system (panels **b**,**c**). Geographical Information Software QGIS 3.18.2 was used ( available at https://qgis.org/downloads/), employing ESPG 4326 (WGS 84) (panels **a**–**c**), Ecuador limits were retrieved from Geographic Military Institute (retrieved from https://sni.gob.ec/geoservicios-ecuador), Urban Growth of Quito was used for Quito limits (retrieved from http://gobiernoabierto.quito.gob.ec/?page_id=1122), Quito’s monitoring sites were retrieved from Secretaría de Ambiente from Quito (http://www.quitoambiente.gob.ec/index.php/generalidades), Road blocks were retrieved from Rcuadorian newscasts (available at https://www.primicias.ec/noticias/sociedad/quito-manifestaciones-vias/ and ) and Satellite image was used from XYZ Tiles service from Google (available at http://mt0.google.com/vt/lyrs=t&hl=en&x={x}&y={y}&x={z}).

Distribution patterns of the concentrations, in μg g^-1^ DW, of the metals found in the *Melaleuca armillaris* leaves, collected at the urban park, housing the most intense protest activities, are presented in Fig. 4. Each of the metals is presented in a separate panel with the range of concentration from minimum to maximum values, depending on metal abundance in the sample. In general, the levels of all metals are higher at points E1 and E7 (see Fig. 4), where most of the visible burn signs were observed during the recollection of the leaf samples. In addition, in those sampling sites tree leaves were profusely covered in soot. Highest levels at those sites were especially observed for Co (Fig. 4a), Cr (Fig. 4b), Zn (Fig. 4c), Al (Fig. 4d), Fe (Fig. 4e), Pb (Fig. 4f), Mg (Fig. 4g) and Ba (Fig. 4h). Those metals are typically used in tire manufacture^27,28,45^. Cu is also known to make part of the composition of tires^27,28^, and its concentration was higher at point E4 (Fig. 4i). At point E4, visible burn signs were not observed at the moment of the sampling. However, it is located just in front of a street where there was a barricade (Fig. 5c) with an intense burning of materials during the protests, mainly tires. At E4 an elevated concentration of K, which is a tracer of biomass burning, was also observed (Fig. 4j). While K is often a good marker of biomass burning, it is also one of the chemical components naturally present in the plants. This might suggest that while at E1 and E7 mostly tires were burned, at E4 there was a mix of pollutants. As previously mentioned, protesters often burn wood to reduce the eye burning due to the tear gas, but also burn tires and other objects to create a dark smoke and block roads (Figure A3, Appendix 1). Finally, site E6 shows increased levels of Mn, Al and Fe, which are metals associated with both natural and road traffic emissions, because they are also components of steel and alloys widely used by the automotive industry^37,46^. Point E6 is near a major road with bus traffic, which could explain the high concentration of these metals accumulating over time^47,48^.

To be able to understand how the concentrations of metals found in an urban park after the protest weight against concentrations in urban areas with different levels of contamination^49^, we compared metal concentrations found in *Melaleuca armillaris* and *Araucaria heterophylla*. The analysis showed a higher capture capacity of the *Araucaria heterophylla* needles in relation to the *Melaleuca armillaris* leaves at point E4 (see Table A2). Table A2 shows that the concentration of Zn, Al, Pb, Fe, Cu and K in the Araucaria heterophylla needles collected at point E4 compared well to the areas with high and moderate vehicular traffic intensity, which are areas considered to have high concentration of pollutants^49^. This, together with the fact that the levels of most of the metals analyzed in the *Melaleuca armillaris* leaves were higher at points where visible burn signs were observed during the recollection of the leaf samples (E1 and E7), could indicate that the concentration of metals increased in this area during the protests due to the burning of materials.

As a result, our study helps understand the importance of the toxicity of urban PM during such events. While overall concentrations of PM_2.5_ seem to have dropped, there are peak concentrations generated by burning different objects that might produce high-level toxicity plumes. It is a quite different situation compared to COVID-19 quarantine regulation improvements on air quality and health seen worldwide^50^. It is known that even low levels of atmospheric particulates, loaded with heavy metals, may build-up in soils, plants and either right away or over time transport to the lungs of urban population and accumulate. It might be of a significant concern, as it has been shown before, that levels of pollution, specifically heavy metals in PM, in developing countries result in much higher concentrations of heavy metals in human matrices (e.g. blood, breast milk, etc.)^51^. Which might cause an extensive range of health conditions, including lung cancer, elevated blood pressure, organ failure (e.g. kidney), bone damage, and developmental and neurobehavioral disorders^52^. A number of studies consistently suggest that the WHO may be under-estimating air pollution impacts in developing countries, especially due to short-term exposure to PM^53,54^. These findings raise a concern for immediate actions to control toxic pollutant peaks in densely populated urban areas.

## Conclusions

In this paper the effect of national protest of 2019 in Ecuador on particulate matter concentrations was estimated. Satellite data indicates that the main changes were observed in capital Quito, where PM_2.5_ levels reduced during the 12-day (October 2–13, 2019) political protest. While regional effect of PM_2.5_ pollution from the Colombian cities and active volcano ash plumes can be detected in this Andean country, the protest had a positive regional impact on environmental quality.

A simple comparison of PM_2.5_ levels before, during and after the strike, confirms the findings of satellite data, these results might be skewed by the changes in meteorological conditions. Thus, the use of meteorology-normalized Machine Learning approach (Artificial Neural Network) estimated daily PM_2.5_ concentrations reduction of − 10.75 ± 17.74% during the strike due to the limitations of traffic activities. The biggest reduction was found in the traffic-busy central area (− 47.84 ± 14.40%), in contrary to other barricade burning areas. It can be concluded that even when most of the anthropogenic activities are seized in an urban area due to the protests, other types of unusual activities might keep worsening the air quality.

Elemental PM analysis of the *Melaleuca armillaris* plant from the central protest action spot—an urban park—shows increased levels of most anthropogenic metals at points with visible burn signs. This is especially observed with Co, Cr, Zn, Al, Fe, Pb, Mg, Ba and Cu, which are metals typically used in tire (common object burned during protest) manufacture. In addition, at one of these points an elevated concentration of a biomass burning tracer K was also observed.

Our study contributes to the understanding of the importance of urban PM toxicity during such unusual events, however, escalating in occurrence. While at the city and regional levels, concentrations of PM decreased, there were peak concentrations generated by burning different objects that might produce high-level toxicity plumes. It might be of a significant concern, as even short-term exposure to toxic PM might cause a wide range of health problems, including lung cancer, elevated blood pressure, organ failure (e.g. kidney), bone damage, and developmental and neurobehavioral disorders. These findings call to not overlook the importance of toxic spikes produced by these events in the context of an overall reduction of air pollution.

## Methods

### Study sites and data analysis

High elevation (2850 m above sea level (m.a.s.l.)) Ecuadorian capital Quito is located between the western and eastern branches of the Andes mountains (Fig. 5a). The Metropolitan District of Quito (DMQ), located on the equatorial line, stretches over a variety of elevations on the side of Pichincha volcano (elev. 4800 m.a.s.l.) and inner Andean valleys (approx. elev. 2300 m.a.s.l.), causing a formation of microclimates ranging in precipitation accumulation^40^. The DMQ has almost 3,000,000 inhabitants^55^ and a rapid growth in motorized fleet running on poor-quality fuels, which considerably affects air quality^39,56^.

In the present investigation of air quality changes due to the political protests in Quito, PM_2.5_ concentration data were taken from the environmental monitoring network of the Secretariat of the Environment of DMQ. Out of nine study sites, distributed across the city, data from seven sites were analyzed: S1-Carapungo (elev. 2660 m.a.s.l, coord. 78°26′50′′W, 0°5′54′′S), S2-Cotocollao (elev. 2739 m.a.s.l, coord. 78°29′50′′W, 0°6′28′′S), S3-Belisario (elev. 2835 m.a.s.l, coord. 78°29′48′′W, 0°11′4.57′′S), S4-Centro (elev. 2820 m.a.s.l, coord. 78°30′36′′W, 0°13′12′′S); S5-Camal (elev. 2840 m.a.s.l, coord. 78°30′36′′W, 0°15′00′′S), S6-Guamani (S5) (elev. 3066 m.a.s.l., coord. 78°33′5′′W, 0°19′51′′S) and S7-Chillos (elev. 2453 m.a.s.l, coord. 78°27′36′′W, 0°18′00′′S) (see Fig. 5b). The air quality and meteorological data were collected through the methods in accordance with the guidelines of the United States Environmental Protection Agency^57^, previously described in detail^39^. The ground-based monitoring data were collected during September 20–October 25, 2019, to include the 12-day periods before, during and after the strike. Daily averages and accumulation (rain only) were processed. In addition, 12-day statistics for some parameters (PM_2.5_, wind speed and relative humidity) were calculated. For daily and 12-day data analysis and visualization, MS Excel and Igor Pro 6.0 (Wavemetrics Inc.) softwares were used.

Additionally, *Melaleuca armillaris* leaves were sampled in the El Ejido Park (Fig. 5c). This park was the hot spot of the protests, and where a large number of materials, such as wood and tires, were burned continuously throughout the strike. The plant samples were taken at seven different points distributed across the urban park (Fig. 5c, points E1–E7). In some of these points, there were obvious signs of burning at the moment of the sampling collection (Fig. 5c, red markers), while in others not (Fig. 5c, magenta markers). It is to be mentioned that, although at the point E4 there were no signs of burning, this point is located just in front of a blockage of the road, where tires were burned, thus we marked it as one of the intense burn sites (see Fig. 5b and c, red markers).

### PM_2.5_ image set

All maps used to illustrate this research are original productions of the authors. For this research, we used the concentration images (μg m^−3^) of PM_2.5_ forecast obtained from the European Centre for Medium-Range Weather Forecasts (ECMWF)^58^, which is based on the use of algorithms and Moderate Resolution Imaging Spectroradiometer (MODIS)^59,60^. The latter provides forecasts of PM_2.5_ behavior. For this study, the Google Earth Engine (GGE) platform was used to provide daily information, with a pixel resolution of 44.5 km^2^. Daily satellite images for 12 days before the protests (20/09/2019–1/10/2019), during the protests (02/10/2019–13/10/2019) and after protests (14/10/2019–25/10/2019) were analyzed for the concentration evolution of PM_2.5_ in the Ecuadorian capital, in addition, rasters from Ecuador and Colombia were downloaded as an indirect area of influence for the analysis of protests in Quito. These data are available at https://developers.google.com/earth-engine/datasets/catalog/ECMWF_CAMS_NRT?hl=en. The visualization was performed using ARCGis PRO software.

### Machine learning modelling

A machine learning (ML) approach was used to quantify the effect of the protests on fine particulate matter concentrations, in seven districts of Quito (see “Satellite PM_2.5_ concentration da” section). Artificial Neural Networks (ANN) were trained to predict business-as-usual PM_2.5_ from hourly measurements of meteorological and temporal variables. Since the models are built from meteorological parameters, the prediction keeps accurate even if unusual climatic conditions occur^41^. This is the reason why this approach is also known as meteorology-normalized modelling^42^. Seven meteorological features were selected: relative humidity, precipitation, temperature, solar radiation, pressure, wind speed and wind direction. The temporal attributes were year day, weekday, hour and trend (date index). Our dataset is composed of 41640 instances, which is suitable for training an ANN. The dataset was split into a training set (1/1/2015–4/1/2019) and a testing set (4/2/2019–10/1/2019). It means that the accuracy of the models was tested in the six months prior to the protests. The Root Mean Squared Error (RMSE) and the Coefficient of Determination (R^2^) were the metrics chosen to assess the performance of the models. To validate a model, the RMSE must be as small as possible, and the R^2^ the closest to 1.

The ANN models were used to estimate the business-as-usual PM_2.5_ concentration. Then, the concentration change was computed from the difference between ML-based business-as-usual and actual value measured during the protests. A specific type of ANN, called Recurrent Neural Network (RNN), was chosen for being especially designed to capture information from sequences, such as time series data. The algorithm implements a recursive method, in which a new state of the model at time *t* is a function of its previous state (*S*_*t-1*_) and the input (meteorological and temporal features) at time *t*, as described in Eq. 1:

1 $$ S_{t} = \, F_{w} \left( {S_{t - 1} , \, X_{t} } \right) $$

where *X*_*t*_ is the input a timestep *t*; *S*_*t*_ is the state at timestep *t*; and *F*_*w*_ is the recursive function. An adaptation of the simple RNN, called Long Short-Term Memory (LSTM), can also be applied for time series estimation of fine particulate matter, in which the prediction is long-term dependent (i.e., the accuracy is highly dependent on the number of states considered). This is the reason why these two types of RNN were tested and evaluated in this study. For each district, the change of concentration was quantified from the method (simple RNN vs LSTM) that provides the most accurate prediction (i.e., the lowest RMSE). All the deep ANN models were developed in Python, using the open-source library Keras.

### Plant description and sampling

In this study, *Melaleuca armillaris* plant was used to help understand the strike PM chemistry. *Melaleuca* is a shrub or small tree (see Figure A4, Appendix 1) genus of 290 species in the myrtle family, Myrtaceae, originating from Australasian region. Their flower generally forms a showy spike resembling a brush, up to 7 cm long, with many flowers generally white^61^. A specimen of the species is deposited at Herbarium QCA and HUTI in Ecuador (collection number Oleas # 1053). All collecting procedures followed the guidelines approved by the Ministerio del Ambiente in Ecuador, described in detail under permit number MAAE-ARSFC-2020–0778. The species was identified by Nora Oleas, using the key at https://plantnet.rbgsyd.nsw.gov.au/cgi-bin/NSWfl.pl?page=nswfl&lvl=gn&name=Melaleuca#11.

*Melaleuca armillaris* leaves were sampled in the El Ejido Park (Fig. 5c) in the morning of 15th of October 2019, less than two days after the strike ended and before the next rain event (Figure A1, Appendix 1). We note that sample collection during the strike was impossible for personal safety due to the aggressive riots between the protesters and the police officers (Figure A3, Appendix A1). The collection of the leaves was carried out following the procedure previously used by the research group^36,37^. Briefly, nearby 20 g of leaves were taken using gloves and kept in polyethylene bags. The samples were taken from the four directions of the trees at a height of about 2 m above the ground. The samples were transported to the laboratory and conserved at 4 °C in the dark until analysis. Part of the samples (about 2 g) were dried in triplicate at 70 ± 2 °C in order to report the metal concentrations on a dry weight.

### Plant sample preparation and analysis

The details about digestion process and quantification of metals in leaves have been fully explained elsewhere^36,37^. In brief, 7 mL of HNO_3_, 2 mL of H_2_O_2_ and 1 mL of H_2_O were added to 0.5 g of fresh leaves and heated at 200 °C during 45 min in a microwave digester (MARS 6 – CEM Corporation). The extraction for each point was performed in triplicate. The samples were filtered followed by adjusting the volume to 25 mL with Milli-Q water, and the concentrations of metals were measured using an Inductively Coupled Plasma-Optical Emission Spectroscopy (ICP-OES, Thermo Scientific iCAP 7000 Series). The metals included in the composition of tires, with given certified values and recoveries in the range of 43.41–91.14%, were analyzed (i.e., Al, Fe, Zn, Pb, Cu, Mg, Co, Ba and Cr). K (% Recovery 78.89) was also quantified, since it is a metal commonly detected during biomass burning. Moreover, more natural metals, such as Ca and Mn (%Recovery 88.02 and 111.01, respectively), were also measured. For the recovery percentage, the certified reference material NIST SRM 1575a—Trace elements in Pine Needles was used. The limits of detection (LOD) were calculated as 3 times the standard deviation of 10 blanks measurements divided by the slope of the analytical curve, while the limits of quantification (LOQ) were calculated similarly by multiplying the standard deviation by 10. The range values for LOD and LOQ were 2.58 × 10^−7^–0.0088 μg g^−1^ and 8.59 × 10^−7^–0.029 μg g^−1^, respectively.

In order to have an idea if the concentrations of metals found in this area were high—since there was no data from before the protest events – an extra sample of Araucaria heterophylla needles was collected at point E4 for the same chemical analysis. The Araucaria heterophylla concentrations of metals in the urban park were then compared with those found in the streets of Quito with a range in vehicular traffic intensity^49^. It is important to compare the concentrations using the same plant species since it has been widely reported in literature that the accumulation capacity of plants for pollutants is species dependent^62,63^.

### Visualization of spatial data

Apart from time series graphs, urban surface level PM_2.5_ evolution was presented in a visual manner using QGIS 3.18 software, employing ESPG 4326 (WGS 84) and EPSG.32717 (WGS84-UTM17 South) coordinate system. Geographical Information Software QGIS 3.18.2 was used (available at https://qgis.org/downloads/), employing ESPG 4326 (WGS 84), Ecuador limits were retrieved from Geographic Military Institute (retrieved from https://sni.gob.ec/geoservicios-ecuador), Urban Growth of Quito was used for Quito limits (retrieved from http://gobiernoabierto.quito.gob.ec/?page_id=1122), Quito’s monitoring sites were retrieved from Secretaría de Ambiente from Quito (http://www.quitoambiente.gob.ec/index.php/generalidades). All this information is available by free public access. Roadblock locations were retrieved from Ecuadorian newscasts (available at https://www.primicias.ec/noticias/sociedad/quito-manifestaciones-vias/, https://www.elcomercio.com/actualidad/bloqueos-escombros-personas-vias-quito.html) and Satellite image was used from XYZ Tiles service from Google (available at http://mt0.google.com/vt/lyrs=s&hl=en&x={x}&y={y}&z={z}).

An Inverse Distance Weighting (distance coefficient p = 2; Grid resolution 110 m) was performed for data interpolation between the monitoring network points.

To present plant chemical analysis in an urban park, QGIS 3.18 software was used. EPSG.32717 (WGS84-UTM17 South) coordinate system was used. An Inverse Distance Weighting (distance coefficient p = 2; Grid resolution 1 m) was performed for data interpolation between the sampling points.

For the visualization of the ECMWF raster of PM_2.5_ the ArcGis PRO software was used, with a resolution of 44 km /pixel, with a WGS84 projection system with ESPG 4326 coordinate system.

## Supplementary Information

Supplementary Information.

## References

[1] Brook RD (2010). Particulate matter air pollution and cardiovascular disease: An update to the scientific statement from the American Heart Association. Circulation.

[2] Pope, C. A., Coleman, N., Pond, Z. A, & Burnett, R. T. Fine particulate air pollution and human mortality: 25+ years of cohort studies. *Environ. Res.*, 108924 (2019). 10.1016/j.envres.2019.108924 31831155

[3] Pope, C. A., & Dockery, D. W. Health effects of fine particulate air pollution: Lines that connect health effects of fine particulate air pollution 2247. vol. 2247. 10.1080/10473289.2006.10464485 (2012). 10.1080/10473289.2006.10464485 16805397

[4] Karagulian F (2015). Contributions to cities’ ambient particulate matter (PM): A systematic review of local source contributions at global level. Atmos. Environ..

[5] Shek DTL (2020). Protests in Hong Kong (2019–2020): A perspective based on quality of life and well-being. Appl. Res. Qual. Life.

[6] Ting T (2020). From ‘be water’ to ‘be fire’: Nascent smart mob and networked protests in Hong Kong. Soc. Mov. Stud..

[7] Brimblecombe P (2020). Street protests and air pollution in Hong Kong. Environ. Monit. Assess..

[8] Dettmer, J. (2019). A year of protest. *Voice of America* (2019). [Online]. https://www.voanews.com/europe/2019-year-protest.

[9] Cerva Cerna D (2020). La protesta feminista en México: la misoginia en el discurso institucional y en las redes sociodigitales. Rev. Mex. Cienc. Polit. Soc..

[10] Rebón J, Ruiz Encina C (2020). Revueltas en y contra el neoliberalismo Argentina, 2001 y Chile, 2019. Sociedad.

[11] Stefanoni, P. ¿Qué pasa en Bolivia? *Nueva Sociedad* (2019). https://nuso.org/articulo/Bolivia-Evo-Morales-Carlos-Mesa-elecciones/. Accessed: 23 Feb 2021.

[12] Espinoza Chapula, A. Reelección presidencial en Bolivia, 2005–2019. Universidad Autónoma de Guerrero (México)) (2020).

[13] Rodríguez Pinzón, É. Colombia 2020: La movilización social como oportunidad y reflejo del cambio. *Analisis Fundacion Carolina* (2020). https://www.fundacioncarolina.es/wp-content/uploads/2020/01/AC-1.20.pdf.

[14] Bastos S, Andrade S (2020). Ecuador, octubre de 2019: “Fue un movimiento de jóvenes, jóvenes indígenas y más". Encartes.

[15] Buben J, Radek A, Kouba K (2020). Nicaragua in 2019: The surprising resilience of authoritarianism in the aftermath of regime crisis. Rev. Cienc. Polít..

[16] BBC News. Renuncia Manuel Merino: la ola de protestas en Perú que dejó dos muertos y 100 heridos y culminó con la dimisión del presidente. *Mundo* (2020). https://www.bbc.com/mundo/noticias-america-latina-54948270.

[17] Sharma AR, Kharol SK, Badarinath KVS (2010). Influence of vehicular traffic on urban air quality—A case study of. Transp. Res. Part D.

[18] Debone D, Ferreira L, Leirião L, Georges S, Khouri E (2020). Urban Climate Air quality and health impact assessment of a truckers’ strike in Sao Paulo state, Brazil: A case study”. Urban Clim..

[19] Demarini, D. M. *et al.* Mutagenicity and chemical analysis of emissions from the open burning of scrap rubber tires. **28**(1), 136–141 (1994). 10.1021/es00050a018 22175842

[20] Bølling, A. K. *et al.* Health effects of residential wood smoke particles: the importance of combustion conditions and physicochemical particle properties. **20**(i) (2009). 10.1186/1743-8977-6-29 PMC2777846 19891791

[21] Singh A (2015). Uncontrolled combustion of shredded tires in a land fi ll e Part 2: Population exposure, public health response, and an air quality index for urban fi res. Atmos. Environ..

[22] Downard, J. *et al.* Uncontrolled combustion of shredded tires in a land fill e Part 1: Characterization of gaseous and particulate emissions. **104**, 195–204 (2015). 10.1016/j.atmosenv.2014.12.059 PMC4316387 25663800

[23] Blaho-Owens, K. Chemical crowd control agents. J. B. T.-E. of F. and L. M. Payne-James, Ed. Elsevier, pp. 319–325 (2005).

[24] Bari A (2011). Characterisation of particulates and carcinogenic polycyclic aromatic hydrocarbons in wintertime wood- fi red heating in residential areas. Atmos. Environ..

[25] Gustafson, P., Johannesson, S., Boman, J., Molna, P., & Barrega, L. Domestic wood burning and PM 2.5 trace elements: Personal exposures, indoor and outdoor levels. **39**, 2643–2653 (2005).

[26] Schmidl, C. *et al.* Chemical characterisation of fine particle emissions from wood stove combustion of common woods growing in mid-European Alpine regions (2007).

[27] . Jimoda, L. A, Sulaymon, I. D., Alade, A. O., Adebayo, G. A. Assessment of environmental impact of open burning of scrap tyres on ambient air quality. *Int. J. Environ. Sci. Technol.* (2017).

[28] Shakya P, Shrestha P, Tamrakar C, Bhattarai P (2006). Studies and determination of heavy metals in waste tires and their impacts of the environment. Pak. J. Anal. Environ. Chem..

[29] Franzosi R (1989). One hundred years of strike statistics: Methodological and theoretical issues in quantitative strike research. ILR Rev..

[30] Ortiz, I., Burke, S., Berrada, M., & Cortes, H. “World protests 2006–2013. *SSRN: Initiative for Policy Dialogue and Friedrich-Ebert-Stiftung New York Working Paper No. 2013* (2014). https://papers.ssrn.com/sol3/papers.cfm?abstract_id=2374098.

[31] Chiquetto JB, Alvim DS, Rozante JR, Faria M, Rozante V, Gobo JPA (2021). Impact of a truck Driver’s strike on air pollution levels in São Paulo. Atmos. Environ..

[32] Meinardi S, Nissenson P, Barletta B, Dabdub D, Rowland FS, Blake DR (2008). Influence of the public transportation system on the air quality of a major urban center. A case study: Milan, Italy. Atmos. Environ..

[33] Basagaña X (2018). Science of the total environment effect of public transport strikes on air pollution levels in Barcelona (Spain). Sci. Total Environ..

[34] Mateos, A. C., Amarillo, A. C., Carreras, H. A., & González, C. M. Land use and air quality in urban environments: Human health risk assessment due to inhalation of airborne particles. **161**, 370–380 (2018). 10.1016/j.envres.2017.11.035 29197278

[35] Henrique P (2015). Chemosphere biomonitoring of metals for air pollution assessment using a hemiepiphyte herb (*Struthanthus flexicaulis*). Chemosphere.

[36] Mancheno T, Zalakeviciute R, González-Rodríguez M, Alexandrino K (2021). Assessment of metals in PM10 filters and Araucaria heterophylla needles in two areas of Quito, Ecuador. Heliyon.

[37] Alexandrino K, Viteri F, Rybarczyk Y, Guevara Andino JE, Zalakeviciute R (2020). Biomonitoring of metal levels in urban areas with different vehicular traffic intensity by using Araucaria heterophylla needles. Ecol. Indic..

[38] Toulkeridis, T. *et al.* Volcanic Ash as a precursor for SARS-CoV-2 infection among susceptible populations in Ecuador: A satellite Imaging and excess mortality-based analysis. *Disaster Med. Public Health Prep.*, pp. 1–37 (2021). 10.1017/dmp.2021.154 PMC8314306 34006342

[39] Zalakeviciute, R., Rybarczyk, Y., Lopez Villada, J., & Diaz Suarez, M. V. Quantifying decade-long effects of fuel and traf fi c regulations on urban ambient PM 2.5 pollution in a mid-size South American city. **9**, 66–75 (2018).

[40] Zalakeviciute R, López-Villada J, Rybarczyk Y (2018). Contrasted effects of relative humidity and precipitation on urban PM2.5 pollution in high elevation urban areas. Sustainability.

[41] Rybarczyk, Y., & Zalakeviciute, R. Assessing the COVID-19 impact on air quality: A machine learning approach. *Geophys. Res. Lett.* **48**(4), e2020GL091202 (2021). 10.1029/2020GL091202 PMC7995168 33785973

[42] Grange SK, Carslaw DC, Lewis AC, Boleti E, Hueglin C (2018). Random forest meteorological normalisation models for Swiss PM10 trend analysis. Atmos. Chem. Phys..

[43] Zalakeviciute R (2020). Drastic improvements in air quality in Ecuador during the COVID-19 outbreak. Aerosol Air Qual. Res..

[44] Kleine Deters J, Zalakeviciute R, Gonzalez M, Rybarczyk Y (2017). Modeling PM2.5 urban pollution using machine learning and selected meteorological parameters. J. Electr. Comput. Eng..

[45] Thorpe A, Harrison RM (2008). Sources and properties of non-exhaust particulate matter from road traffic: A review. Sci. Total Environ..

[46] Fujiwara F, Jiménez R, Dawidowski L, Gómez D (2011). Spatial and chemical patterns of size fractionated road dust collected in a megacitiy. Atmos. Environ..

[47] Zalakeviciute, R., Alexandrino, K., Rybarczyk, Y., Debut, A., Vizuete, K., & Diaz, M. Seasonal variations in PM10 inorganic composition in the Andean city. *Sci. Rep.* **10**(1) (2020). 10.1038/s41598-020-72541-2 PMC7550351 33046746

[48] Zalakeviciute, R., Rybarczyk, Y., Granda-Albuja, M. G., Suarez, M. V. D., & Alexandrino, K. Chemical characterization of urban PM10 in the tropical andes. *Atmos. Pollut. Res.* (2020).

[49] Alexandrino K, Viteri F, Rybarczyk Y, Ernesto J, Andino G, Zalakeviciute R (2020). Biomonitoring of metal levels in urban areas with different vehicular traffic intensity by using Araucaria heterophylla needles. Ecol. Indic..

[50] Liu F, Wang M, Zheng M (2021). Effects of COVID-19 lockdown on global air quality and health. Sci. Total Environ..

[51] Hadei M (2021). A systematic review and meta-analysis of human biomonitoring studies on exposure to environmental pollutants in Iran. Ecotoxicol. Environ. Saf..

[52] World Health Organization. Health risks of heavy metals from long-range transboundary air pollution (2007)

[53] Hadei M (2020). Burden of mortality attributed to PM2.5 exposure in cities of Iran; contribution of short-term pollution peaks. Atmos. Environ..

[54] Hopke PK (2018). Spatial and temporal trends of short-term health impacts of PM2.5 in Iranian cities; a modelling approach (2013–2016). Aerosol Air Qual. Res..

[55] Instituto Nacional de Estadísticas y Censos (INEC). Proyecciones Poblacionales. *Poblacion* (2013). https://web.archive.org/web/20131018060046/https://www.ecuadorencifras.gob.ec/proyecciones-poblacionales/.

[56] Zalakeviciute, R., Bastidas, M., Buenaño, A., & Rybarczyk, Y. A traffic-based method to predict and map urban air quality. *Appl. Sci.* **10**(6) 2020.

[57] Secretaría de Ambiente. Secretaria de Ambiente: Informe Final Inventario de Emisiones de Contaminantes Criterio, DMQ 2011. p. 53 (2014).

[58] Vu BN (2019). Developing an advanced PM2.5 exposure model in Lima, Peru. Remote Sens..

[59] Lyapustin A, Wang Y, Korkin S, Huang D (2018). MODIS collection 6 MAIAC algorithm. Atmos. Meas. Tech..

[60] Benedetti A. *et al.* Aerosol analysis and forecast in the European Centre for Medium-Range Weather Forecasts Integrated Forecast System: 2. Data assimilation. *J. Geophys. Res. Atmos. ***114**(D13) (2009).

[61] Brophy, J. J., Craven, L. A., & Doran, J. C., *Melaleucas: their botany, essential oils and uses*. School of Chemistry, University of New South Wales, Sydney, New South Wales 2052, Australia. (2013).

[62] Solgi E, Keramaty M, Solgi M (2020). Biomonitoring of airborne Cu, Pb, and Zn in an urban area employing a broad leaved and a conifer tree species. J. Geochem. Explor..

[63] Wannaz ED, Carreras HA, Pérez CA, Pignata ML (2006). Assessment of heavy metal accumulation in two species of Tillandsia in relation to atmospheric emission sources in Argentina. Sci. Total Environ..

