# Author Correction: The effect of national protest in Ecuador on PM pollution

**DOI:** 10.1038/s41598-021-98465-z

**Published:** 2021-09-14

**Authors:** Rasa Zalakeviciute, Katiuska Alexandrino, Danilo Mejia, Marco G. Bastidas, Nora H. Oleas, Diana Gabela, Phuong Ngoc Chau, Santiago Bonilla-Bedoya, Valeria Diaz, Yves Rybarczyk

**Affiliations:** 1 Grupo de Biodiversidad Medio Ambiente y Salud (BIOMAS), Universidad de Las Américas, Calle José Queri y Av. de los Granados / Bloque 7, 170125 Quito, EC Ecuador; 2 Facultad de Ciencias Químicas de La Universidad de Cuenca, Cuenca, Ecuador; 3 Centro de Estudios Ambientales (CEA) de la Universidad de Cuenca, Cuenca, Ecuador; 4 Centro de Investigación de la Biodiversidad y Cambio Climático (BioCamb) e Ingeniería en Biodiversidad y Recursos Genéticos, Facultad de Ciencias de Medio Ambiente, Universidad Tecnológica Indoamérica, Machala y Sabanilla, 170301 Quito, EC Ecuador; 5 Faculty of Data and Information Sciences, Dalarna University, 791 88 Falun, Sweden; 6 Research Center for the Territory and Sustainable Habitat, Universidad Tecnológica Indoamérica, Machala y Sabanilla, 170301 Quito, Ecuador; 7 Secretariat of the Environment, Quito, Ecuador

Correction to: *Scientific Reports* 10.1038/s41598-021-96868-6, published online 02 September 2021

In the original version of this Article Rasa Zalakeviciute, Katiuska Alexandrino, Danilo Mejia, Marco G. Bastidas, Nora H. Oleas, Diana Gabela, Phuong Ngoc Chau, Santiago Bonilla-Bedoya & Yves Rybarczyk were incorrectly affiliated with ‘Secretariat of the Environment, Quito, Ecuador’.

In addition, Valeria Diaz was incorrectly affiliated with ‘Research Center for the Territory and Sustainable Habitat, Universidad Tecnológica Indoamérica, Machala y Sabanilla, 170301, Quito, Ecuador’.

The correct affiliations are listed below:

1. Grupo de Biodiversidad Medio Ambiente y Salud (BIOMAS), Universidad de Las Américas, calle José Queri y Av. de los Granados / Bloque 7, 170125, Quito, EC, Ecuador
  Rasa Zalakeviciute, Katiuska Alexandrino, Marco G. Bastidas & Diana Gabela
2. Facultad de Ciencias Químicas de La Universidad de Cuenca, Cuenca, Ecuador
  Danilo Mejia
3. Centro de Estudios Ambientales (CEA) de la Universidad de Cuenca, Cuenca, Ecuador
  Danilo Mejia
4. Centro de Investigación de la Biodiversidad y Cambio Climático (BioCamb) e Ingeniería en Biodiversidad y Recursos Genéticos, Facultad de Ciencias de Medio Ambiente, Universidad Tecnológica Indoamérica, Machala y Sabanilla, 170301, Quito, EC, Ecuador
  Nora H. Oleas
5. Faculty of Data and Information Sciences, Dalarna University, 791 88, Falun, Sweden
  Phuong Ngoc Chau & Yves Rybarczyk
6. Research Center for the Territory and Sustainable Habitat, Universidad Tecnológica Indoamérica, Machala y Sabanilla, 170301, Quito, Ecuador
  Santiago Bonilla-Bedoya
7. Secretariat of the Environment, Quito, Ecuador
  Valeria Diaz

The original Article has been corrected.

