# Supplementary material for: The effect of national protest in Ecuador on PM pollution
Source: Sci Rep. 2021 Sep 2;11:17591. doi: 10.1038/s41598-021-96868-6 (PMC8413373; doi:10.1038/s41598-021-96868-6)
Supplement: Supplementary file 1 — Supplementary Information. [file 41598_2021_96868_MOESM1_ESM.docx]

Appendix 1


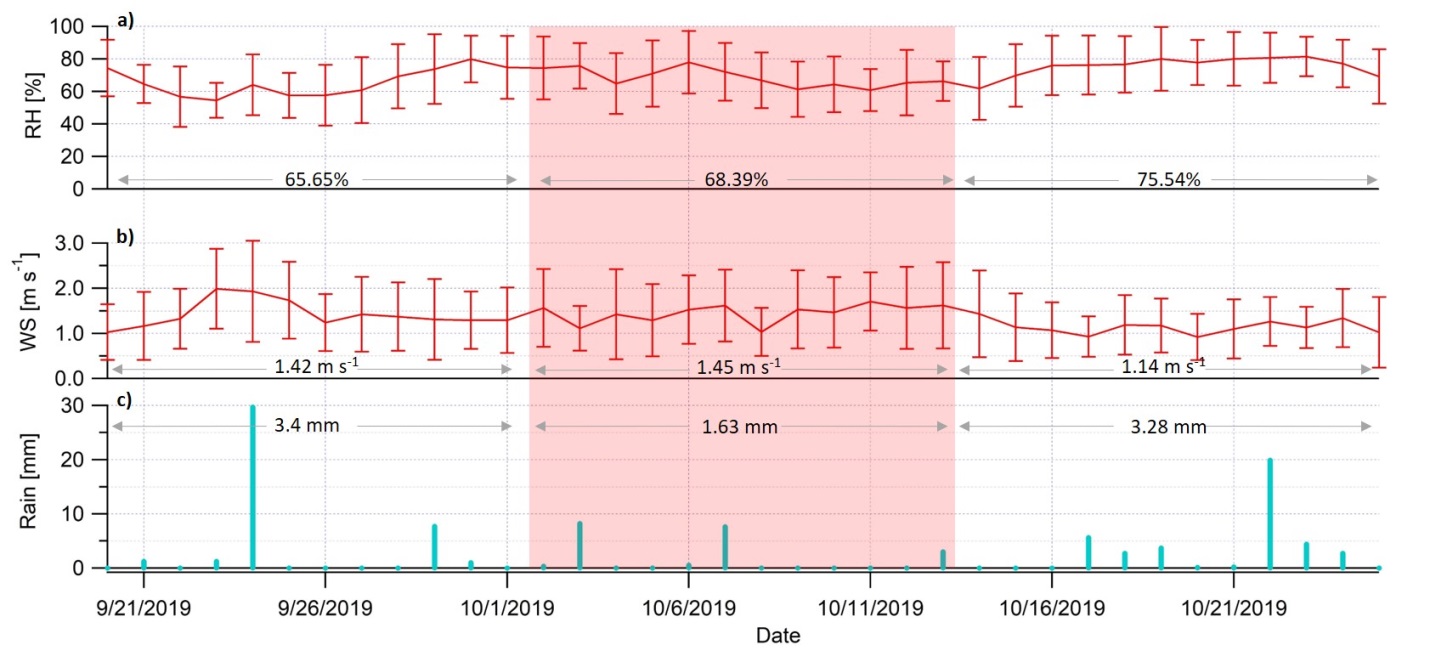


**Figure A1.** Meteorological variables before (9/20/2019-10/01/2019), during (10/02/2019-10/13/2019) and after (10/14/2019-10/25/2019) the strike in the central S3-Belisario site of Quito, Ecuador. 12-day average (panel a: relative humidity (RH); and panel b: wind speed (WS)) and cumulative (panel c: 24-hour rain accumulation) values for each period.


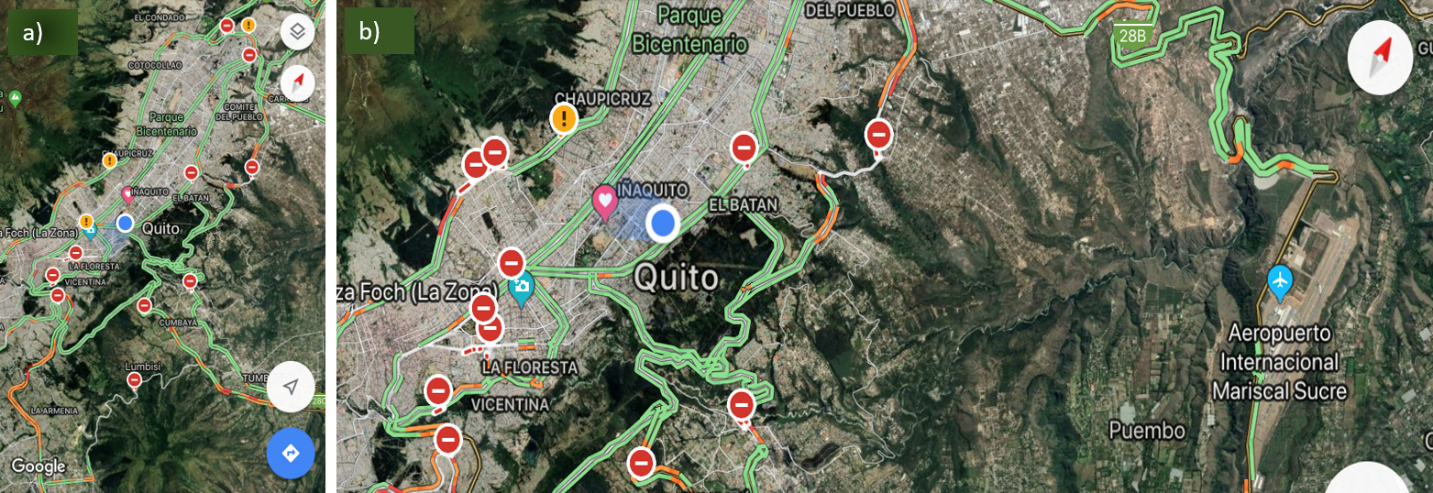


**Figure A2.** Road closures during the protests in Quito Ecuador, (a print screen of Google Maps Traffic application on 13^th^ of October 2019), showing that all the access to the city was cut off by barricades.

**
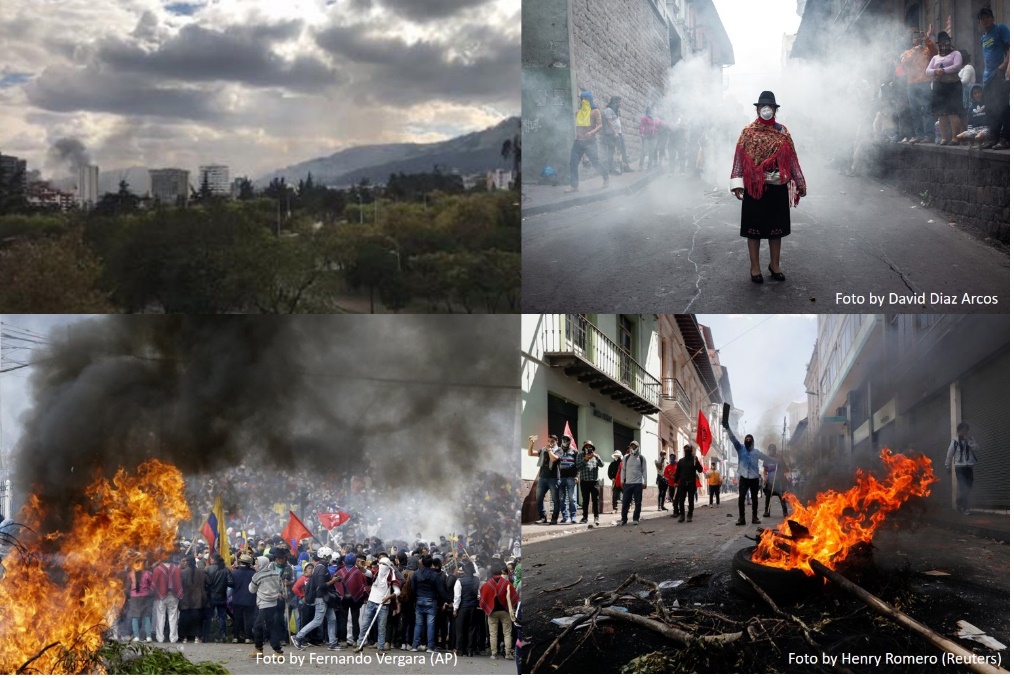
**

**Figure A3.** Compilation of images of the national Ecuadorian protest of 2019. Fotos by different authors (<https://elpais.com/elpais/2019/10/09/album/1570637826_641314.html#foto_gal_18>; https://noticias.utpl.edu.ec/david-diaz-arcos-un-exponente-del-fotodocumental-ecuatoriano-con-proyeccion-internacional).


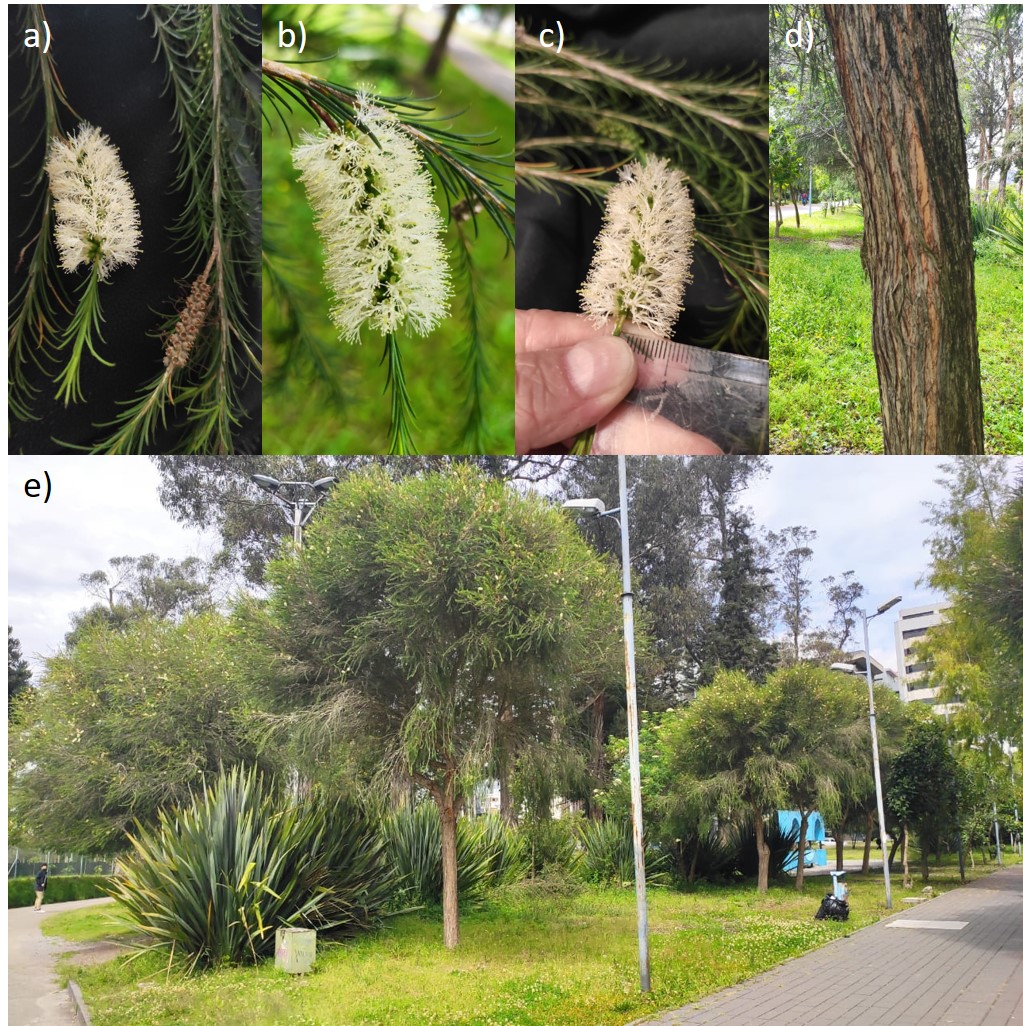


**Figure A4.** *Melaleuca Armilaris*: a) a flower and a fruit; b) a flower and leaves; c) flower size to scale; d) a trunk; e) a complete tree in an urban park of Quito, Ecuador.

**Table A1.** Evaluation of models’ accuracy for different sites. The values in bold indicate which model performs the best for each district, where RMSE stands for root mean square error, LSTM stands for Long- Short-Term Memory and RNN stands for Recurrent Neural Network.

| Site | Statistics | LSTM | Simple RNN |
| --- | --- | --- | --- |
| S1-Carapungo | RMSE | **11.634** | 11.731 |
|  | R2 | **0.234** | 0.221 |
| S2-Cotocollao | RMSE | 9.661 | **9.659** |
|  | R2 | 0.194 | **0.195** |
| S3-Belisario | RMSE | **9.875** | 10.151 |
|  | R2 | **0.255** | 0.213 |
| S4-Centro | RMSE | 10.899 | **10.378** |
|  | R2 | 0.039 | **0.129** |
| S5-Camal | RMSE | **12.581** | 12.95 |
|  | R2 | **0.219** | 0.172 |
| S6-Guamani | RMSE | **15.643** | 16.081 |
|  | R2 | **0.12** | 0.07 |
| S7-Chillos | RMSE | **7.308** | 7.638 |
|  | R2 | **0.193** | 0.118 |

Table A2. Comparison of the concentrations of metals found in the *Callistemon citrinus* leaves and *Araucaria heterophylla* needles recollected at point E4 in the El Ejido Park with those found in the *Araucaria heterophylla* needles recollected at areas with high, moderate and low vehicular traffic intensity in the city of Quito (Alexandrino et al., 2020)

|  | Mg | Zn | Al | Pb | Fe | Cu | Ba | Cr | Co | Ca | Mn | K |
| --- | --- | --- | --- | --- | --- | --- | --- | --- | --- | --- | --- | --- |
| *Callistemon citrinus leaves* |  |  |  |  |  |  |  |  |  |  |  |  |
| Point E4^a^ | 1497.47 | 28.24 | 231.05 | 0.79 | 300.47 | 13.99 | 29.75 | 0.12 | 0.12 | 6093.79 | 57.88 | 8192.64 |
| *Araucaria heterophylla* needles |  |  |  |  |  |  |  |  |  |  |  |  |
| Point E4^a^ | 2223.22 | 40.13 | 642.48 | 2.29 | 1013.63 | 17.14 | 75.74 | 1.68 | 0.39 | 18570.59 | 149.54 | 8056.00 |
| High vehicular traffic intensity^b^ | 3283.60 | 60.43 | 938.01 | 3.22 | 1084.92 | 19.12 | 203.81 | 7.16 | 0.74 | 24906.76 | 1250.95 | 6028.36 |
| Moderate vehicular traffic intensity^b^ | 3290.95 | 42.58 | 732.34 | 1.32 | 646.25 | 8.86 | 112.00 | 3.75 | 0.54 | 24742.86 | 224.49 | 11417.20 |
| Low vehicular traffic intensity^b^ | 3373.83 | 28.52 | 530.92 | 0.18 | 424.50 | 4.38 | 111.56 | 1.90 | 0.48 | 24925.19 | 692.71 | 8484.26 |

^a^ Present work

^b^ Alexandrino, K., Viteri, F., Rybarczyk, Y., Ernesto, J., Andino, G., & Zalakeviciute, R. (2020). Biomonitoring of metal levels in urban areas with different vehicular traffic intensity by using Araucaria heterophylla needles. *Ecological Indicators*, *117*(March), 106701. https://doi.org/10.1016/j.ecolind.2020.106701
